# Children’s Health Lifestyles and the Perpetuation of Inequalities

**DOI:** 10.1177/00221465241255946

**Published:** 2024-06-19

**Authors:** Stefanie Mollborn, Jennifer A. Pace, Bethany Rigles

**Affiliations:** 1 Stockholm University, Sweden; 2 University of Colorado Boulder, Boulder, CO, USA; 3 U.S. Census Bureau, Washington, DC, USA; 4 Good Nutrition Ideas, Eugene, OR, USA

**Keywords:** childhood, family, health behavior, health lifestyle, inequality, parenting

## Abstract

Health lifestyles are a well-theorized mechanism perpetuating health and social inequalities, but empirical research has not yet documented crucial aspects: (1) health lifestyles’ collective nature or content beyond behaviors and (2) how people choose among available lifestyles in their social contexts. We conducted interviews, observations, and focus groups with families in two middle- to upper-middle-class communities. Contemporary class-privileged parenting involves constructing an individualized health lifestyle reliant on an expansive understanding of health and composed of parents’ identities and narratives, children’s health behaviors and identity expressions, and community norms. Children’s predominant health lifestyles in our sample vary by focus on parent versus child identity expression and on future achievements versus present well-being. Parents expect health lifestyles to influence future socioeconomic attainment and health inequalities. Understanding how health lifestyles encompass more than behaviors and are locally contextualized and how people choose them within structural constraints can inform research and policy.

As U.S. health disparities widen and the intergenerational transmission of advantage in families and communities strengthens, increasing attention is being paid to the processes underlying these trends. Of particular interest is early life, when children are influenced by previous generations in ways that will be consequential for decades to come. Sociological research has pinpointed the shaping of children’s everyday lives by parents in ways that foster children’s long-term health and socioeconomic well-being as one important mechanism of the intergenerational transmission of advantage. Notions of children as agents in their own lives, rather than passive, innocent receptacles of socialization (see Pugh 2014), and research on the importance of local norms and institutions (Brown-Saracino 2015) contextualize families within communities as a site for reproducing inequalities.

As scholars are increasingly emphasizing, the concept of “health lifestyles” is useful for understanding how social and health inequalities play out in individuals’ everyday lives (Cockerham 2005; Krueger, Bhaloo, and Rosenau 2009; Weber [1922] 1978). Cockerham (2005:55) defined health lifestyles as “collective patterns of health-related behavior based on choices from options available to people according to their life chances.” Understandings of health lifestyles in early life and as part of the intergenerational reproduction of inequalities are still nascent. Furthermore, although a long theoretical tradition suggests that multiple lifestyle options are available to people with similar social class, most research has treated class-based parenting and its implications for children as relatively monolithic within class (e.g., Lareau 2011). With a health lifestyles approach, scholars can articulate variation within social categories and multilevel conceptualizations of contexts to understand how children’s everyday lives and health are shaped by parents, children, and communities in ways that may reinforce future inequalities—a phenomenon about which not enough is yet known in childhood (Pugh 2014).

Empirical work on health lifestyles lags behind theory in crucial ways. Despite a growing consensus that lifestyles likely extend beyond behaviors, extant (mostly quantitative) research has measured only health behaviors. Furthermore, although it has long been understood that health lifestyles are collective (Cockerham, Rütten, and Abel 1997; Frohlich and Potvin 1999), empirical research has examined them in individuals. Importantly, health lifestyles are theorized to be contextually specific (Cockerham et al. 2004), but almost all research has used national or geographically dispersed samples. Finally, Cockerham (2005:61) theorized, but empirical work has not yet shown, that people “align their goals, needs, and desires with their probabilities for realizing them and choose a lifestyle according to their assessments of the reality of their resources and class circumstances.” This “interplay between life choices and life chances” (Cockerham 2005:60), in which people choose lifestyles from among available options, is fundamental to health lifestyles theory but has not been empirically documented. We seek to address these gaps through a study that situates families within community collectivities and creates space for new understandings of health, health behaviors, and health lifestyles to arise from the data.

This study’s goals are to (1) articulate an inductive model of the components of children’s health lifestyles in two middle- to upper-middle-class communities and (2) identify predominant health lifestyles for community children, how they are chosen, and potential longer-term implications. Our qualitative interview, observational, and focus group data from families with elementary-aged children show that through health lifestyles, parents attempt to shape children’s everyday lives in multifaceted ways that they imagine will affect long-term well-being. Children’s health lifestyles are distinct from adult lifestyles because they blend parent and child identity expression and because of the salience of later life for understanding how and why they form.

We articulate an inductively derived theoretical model of the components of children’s health lifestyles and describe their predominant types in study communities. As Cockerham (2023) and others (e.g., Krueger et al. 2009; Mollborn and Modile 2022) have foreshadowed, this model integrates with and expands the standard definition of health lifestyles beyond health-related behaviors to include understandings of health and health-related norms, narratives, and identities. Although we rely primarily on interviews, multimethod data incorporating observed behavior, private narratives, and public talk document parental identity work, community structures and normative processes, and child identity expression. Through health lifestyles, parents, schools, and communities together influence children’s behaviors, identities, and futures. Health lifestyles are thus a pathway through which social and health advantages persist across lives and generations.

## Background

### The Transmission of Advantage in Early Life

U.S. socioeconomic inequalities have been increasing, and intergenerational class mobility lags behind that of peer countries (Saez 2008). Life expectancy is falling (Muennig et al. 2018), and links between socioeconomic status and health are tightening (Masters, Hummer, and Powers 2012). Two implications of these trends motivate health lifestyles research. First, strong processes transmit social advantage and disadvantage across lives and generations. Lifestyle factors are important for understanding socioeconomic disparities in later health (Puka et al. 2022). Second, socially advantaged parents are experiencing increasing pressures to preserve children’s socioeconomic and health advantages to avoid worsening circumstances among the disadvantaged (Link and Phelan 1995; Nelson 2010). Alongside structural phenomena, such as the intergenerational transmission of wealth and persistent residential segregation, research has focused on family processes—including health-related parenting (Augustine, Prickett, and Kimbro 2017; Christensen 2004)—to understand intergenerational replications of social advantage. Indeed, both healthy behaviors and socioeconomic advantage are increasingly being consolidated in families (Maralani and Portier 2021). The life course perspective, including theories on accumulation and transmission of advantage, often grounds this work (Elder 1994).

### Health Lifestyles

Contemporary intensive parenting and child identity development are manifested in health lifestyle formation. Although children are infrequently addressed, a long-standing literature has examined individuals’ lifestyles as manifestations of social class and other conditions. A *lifestyle* is an individual’s collection of everyday behaviors, expressing group-based identity, regulated by group norms, and constrained by social structures (Cockerham 2013). Social class results in a specific set of lifestyle options being available, and individuals choose among them as an expression of group membership (Weber [1922] 1978). Cockerham (2005) and others (e.g., Frohlich and Potvin 1999; Mollborn et al. 2014) have focused specifically on *health lifestyles*, defined previously, as a core domain of lifestyles. Previous research on health lifestyles focused on adults can be extended to children. Children have relatively less control over their health lifestyles but exercise agency and express identities in conjunction with parental control (Pugh 2014).

National quantitative studies measuring behaviors abound, but little research examines health lifestyles in specific contexts or collectivities (Cockerham 2023). One quantitative study found that health lifestyle behaviors cluster in localized ways (Lee et al. 2015), and another mapped health lifestyles in two schools (adams et al. 2021). A qualitative study found that Scottish adults understood health lifestyles as reflecting identity and lived experience (McGarrol 2020), and another contrasted Norwegian youths’ health lifestyle formation by class standing (Eriksen et al. 2024). Research has not empirically documented aspects of health lifestyles beyond behaviors or how people choose them based on locally available options.

Health lifestyles are not just a resource for future health but also a cultural symbol that itself generates inequalities (Mollborn, Lawrence, and Saint Onge 2021). Korp (2008) emphasized the symbolic power of “healthy” lifestyles as both manifestations of inequality and phenomena that create inequality. Shared notions of “healthy” or “unhealthy” lifestyles legitimize some behaviors and delegitimize others. Lifestyles are an effective form of class distinction (Abel 2008; Bourdieu 1986a), especially because of the increasing moral value being placed on health (Luna 2019). Indeed, laypeople consider “healthy” lifestyle behaviors crucial to well-functioning families (Williamson et al. 2018). Because parenting, like health, has strong moral dimensions (Shirani, Henwood, and Coltart 2012), children’s health lifestyles create class distinctions (Mollborn, Rigles, and Pace 2021). Class-advantaged parents use food to teach classed values about discipline and enact distinction, linking narratives and behaviors (Elliott and Bowen 2018; Fielding-Singh 2019; Wills et al. 2011). These processes have longer-term implications, as Eriksen et al. (2024:149) wrote: “The dense integration of health lifestyle and family life instils a rigorous health orientation in the upper-class child’s habitus, a bodily disposition for health practices equipping them to live—and to wish for—a healthy lifestyle now and as they grow older.”

Conceptualizing lifestyles as a blend of structure and agency (Cockerham 2005), we find that children’s health lifestyles are rooted in parents’ expansive understandings of health, which blur boundaries across physical and psychological well-being, social integration, and academic achievement (Pace, Mollborn, and Rigles 2022; Warner 2010). They are broader than researchers’ implicit understandings of health when operationalizing health lifestyles. Health lifestyles encompass children’s behaviors (including traditional health behaviors and others linked to wider conceptualizations of health, such as socializing and doing homework). Although content likely varies, norms in the communities that we studied prescribe that children’s health lifestyles must reflect substantial parental identity investments and coherent parenting narratives articulating the lifestyle’s benefits for health, well-being, and future success. Children’s identity expression is also dictated by community norms. Health lifestyles create advantages for children while simultaneously reaffirming parents’ advantaged class standing. Health lifestyles likely have implications for socioeconomic attainment, health, and future lifestyles and identities, fueling the intergenerational perpetuation of inequalities.

### Parenting and Inequalities

The literatures on health lifestyles and parenting converse infrequently, and this study exemplifies how they can inform each other. Parenting practices reproduce advantages, instilling cultural, social, and human capital and setting children up for success or failure in interactions with social institutions (Bourdieu 1986b; Lareau 2011) in what parents view as an uncertain world (Nelson 2010). Structural opportunities heavily constrain these practices. Class-advantaged parents leverage resources to select structures considered beneficial for children, such as residential areas, child care, and schools (Augustine, Cavanagh, and Crosnoe 2009; Lareau, Evans, and Yee 2016; Mirowsky and Ross 2015). Beyond “status safeguarding” (Milkie and Warner 2014) via institutions, resource-advantaged parents typically raise children in ways that intensify their likelihood of future success. Schneider, Hastings, and LaBriola (2018) identified larger class disparities in parental financial investments in children in states with higher income inequality, driven by increases among highest income parents.

This exemplifies “intensive parenting,” a strategy in which advantaged parents, especially mothers, leverage abundant resources to instill cultural capital, health, and educational benefits in children (Bourdieu 1986b; Hays 1996; Shirani et al. 2012). Analyses of mothers’ time investments with children suggests that they matter for academic and behavioral outcomes (Fomby and Musick 2018). Calarco (2014) found that parents’ strategies represented deliberate mobilization of class privilege. A backdrop of growing economic insecurity heightens parents’ sense of risk (Cooper 2014).

Group-based identities (Stets and Burke 2000) shape parenting. Collett, Vercel, and Boykin (2015) articulated parenting identity processes for fathers. A parent has a socially influenced identity standard for a “good parent” and seeks to align their parenting behaviors with the standard to minimize discrepancies and verify their identity, reducing negative emotions and social judgments and increasing positive ones. The more flexible the identity standard is, the more leeway there is to avoid negative repercussions. Tsushima and Burke (1999) emphasized the importance for parenting identity of aligning parenting tasks such as managing children’s time with abstract goals like fostering autonomy. Resources facilitate this alignment. Children’s identity expression can also intervene to complicate parents’ efforts (Chin and Phillips 2004). Pugh (2009) found that children pursued goals such as engaging with popular culture and being independent from adults’ control, which often conflicted with adults’ goals. Expanding research on agency to include often overlooked groups like children is important for life course theory (Landes and Settersten 2019).

Health is not directly addressed in much parenting literature, but some studies incorporate it. Intensive parenting among socially advantaged parents can be “individualist parenting” (Reich 2016), reflecting a parent’s vision for the child’s future in ways that are guided by experts but do not always follow standardized recommendations. This can sometimes result in advantaged parents making child health decisions not driven by medical interpretations of the child’s best interests (King, Jennings, and Fletcher 2014; Reich 2016).

## Data and Methods

### Data and Procedures

To understand the empirical scope of health lifestyles, this study’s design was different from typical health lifestyle approaches that focus on a specific set of physical health-related behaviors and their frequencies. We sought to collect qualitative data on children’s health lifestyles, avoiding imposing preexisting notions around what constitutes health and health behaviors for parents, what children’s health lifestyles consist of, and whether parents talk about health lifestyles. Recruitment materials said the study was about “parents, kids, and well-being.” Data collection strategies, procedures, and instruments were refined through pilot research. The data combined in-home family observations, parent interviews and focus groups, and key informant interviews in two neighboring middle-class communities in the U.S. West—“Greenville” and “Springfield”—from September 2015 to May 2016. (All names and some potentially identifying details have been altered.) Our primary data source was 55 parent interviews: 35 with parents who also participated in a home observation (N = 30 families; in 5 families, both parents requested to be interviewed together) and 20 with parents who only participated in an interview. We conducted six focus groups (three for each community), including 21 parents (some of whom had also done interviews and/or observations). The nine key informants interacted with families in the local area (e.g., sports coaches, pediatricians, teachers). The 30 observation families (typically observed from the end of school to the start of the bedtime routine on one school night) included a fourth or fifth grader age 9 to 11. Parents in other interviews and focus groups had at least one elementary-age child. We chose these ages because family influences are still substantial but have been joined by peers, school, and child agency.

We abductively revised our study design in response to emergent evidence and methodological considerations (Timmermans and Tavory 2012), changing our sampling strategy to better balance community data collection and create community-specific parent focus groups. Our broad-based recruitment strategies were designed to diversify the sample (Lofland and Lofland 2006). Rather than relying primarily on snowball sampling, we recruited participants online and through local parenting email listservs, personal contacts, referrals, and public flyers. A few participants recommended the study to others. When we reached 10 parent interviews from a school, we stopped collecting data from its families. The resulting nonrepresentative sample was sociodemographically varied, included many neighborhoods and social networks, and incorporated families from 23 elementary schools and homeschoolers.

Our data collection team included one faculty member and two graduate students (all White women) and three undergraduates (an African American woman, an Asian American man, and a White man). Families received $200 for a home observation with interview, and other participants received $50. The study received institutional review board approval. The faculty member or graduate student conducted interviews and focus groups, which were audio recorded and transcribed. The semistructured interviews started with questions that did not prompt about health, regarding the child’s daily routine, how parents navigate the child’s preferences, how their child’s life compares to their own childhood, what parenting messages they try to convey, and what parenting is like in their community. Parents’ strong focus on health emerged unprompted in these sections. Later, we prompted about health, including how parents define “health” and “well-being,” what shapes children’s health, and so on. Observations were conducted by either the faculty member or a graduate student and an undergraduate. Observers followed different family members in different spaces, yielding two sets of field notes for many interactions and one for others.

Parent participants’ average age was 43, and 80% were mothers. Seventy-seven percent were married, 17% were divorced/separated, 4% were single, and one parent was widowed. Eighty-six percent of parents identified as White, 8% identified as Asian American, and 6% identified as Latino; a substantial minority were foreign-born. Children were 2 to 15 years old, with most in fourth or fifth grade. Based on reported parent and partner education and occupation and housing quality in the family observations, we coded 59% of families as upper-middle class, 29% as middle-class or mixed (e.g., higher education but lower income), and 12% as working class or poor. Most parents had grown up in a similar social class as measured by parental occupations, but some were class-mobile.

### Field Sites

The study’s communities, both midsized cities in the same large metropolitan area (U.S. Census Bureau 2017), were middle- to upper-middle-class. They were demographically quite similar, with median household incomes close to the state average and high proportions of residents identifying as White. Middle-class Springfield was more socioeconomically and ethnically diverse than upper-middle-class Greenville; its median housing value was half as high, and half as many residents had a bachelor’s degree (U.S. Census Bureau 2017). Both communities had unusually high rates of positive health behaviors and low obesity rates and were located in a geographic region that was considered a politically liberal health mecca attracting highly educated new residents.

### Analysis

Electronic copies of transcriptions and field notes were manually coded using NVivo qualitative software and summary spreadsheets. Analysis of themes reported here was inductive: We had no a priori expectation about what children’s health lifestyles consisted of, how health or health behaviors were defined, or how they would vary. We analyzed observational field notes together with parent interviews to compare personal accounts to observed behaviors, allowing themes to arise organically and coding for some predetermined themes. People’s public and private accounts and behaviors are often inconsistent in sociologically meaningful ways (Swidler 2001). Because the communities’ health lifestyles were similar even though distribution and degree varied, we combined the communities here.

We viewed the interviews and focus groups as opportunities for participants to actively construct narratives (Holstein and Gubrium 1995). Through narratives situated in specific social contexts, people construct identities, justify actions, and manage others’ impressions (Swidler 2001). Narratives, which shed light on norms, individual and group identities, and inequalities, turned out to be an important aspect of children’s health lifestyles. Our goal was not to adjudicate whether parenting, specific health lifestyles, or their consequences are good or bad.

## Results

Many parents were familiar with the idea of a healthy lifestyle, and there was some unprompted use of the term. But mostly parents broadly articulated “the way we raise them” (in Laura’s words), often with eloquent narratives, identity statements, and nuanced understandings of health and community norms underlying the behavioral routines they carefully fostered and repeatedly linked to health and well-being. Grounded in these data, we inductively modeled the components of children’s health lifestyles as articulated by parents and described prevalent types in the study communities.

### Components of Children’s Health Lifestyles

We found that children’s health lifestyles combine behavioral and nonbehavioral aspects. Dan, a White, upper-middle-class Springfield father, illustrates this complexity when describing 11-year-old Brittany’s typical weekday routine:

She gets to sleep in. She gets to get a nice homemade breakfast. We walk to school. I personally—Mary [Brittany’s stepmother] and I both feel—it’s a much better life for a child to get a good, full night’s sleep and start the day with a good breakfast. Have some family time . . . organic, cage free, all that good stuff.

In representing Brittany’s everyday lifestyle, Dan makes health salient and blends behavioral routines with narratives, representing his parent identity, that underlie and justify them. Dan continues, “Mary and I are both—I will call it old-fashioned—but we eat dinner together. I don’t answer the phone; neither of us will answer the phone at dinnertime. So we talk during dinner, and we spend a lot of quality time together, not just being under the same roof.” Mealtime behaviors express his and Mary’s “old-fashioned” identities. Dan implicitly distinguishes his family’s lifestyle from his idea of a typical modern family that he believes eats separately.

Dan continues with a parenting narrative on boundary setting, which he links to well-being throughout his interview.

And ever since Brittany was really little, I’ve always been—what’s the word? Fanatical? I think kids do better with consistency, and you can also add into that, boundaries. So she’s always had a set bedtime, which has obviously gotten a little later as she’s gotten older. . . . And I think all human beings operate better, function better with a routine.

Dan does repeated narrative work to distinguish his parenting favorably from that of other parents, whom he views as overly hands-off. He also acknowledges the importance of community for facilitating his desired health lifestyle: “One of the reasons I bought this house is, it’s close to the elementary school, it’s close to middle school, it’s close to the high school. So since she’s an only child, I wanted her to grow up with a lot of friends in her neighborhood that she would get to know all through school.” Mary and Dan organize their work schedules and exercise time to maximize interaction with Brittany. We observed rooms dedicated to her hobbies. Manifesting Dan and Mary’s parenting identities and representing a broad understanding of health from sleep and diet to family interactions and peer contact, Brittany’s everyday routine is carefully curated to create a health lifestyle that they believe sets her up for future success.

Reflecting this example, Table 1 describes our inductively derived model of the components of children’s health lifestyles. As contemporary health lifestyles theory posits (Cockerham 2023), they combine health behaviors and multiple nonbehavioral aspects. Qualitative research on young adults has suggested that “not just health behaviors, but identities, narratives, norms, and understandings of health [are] core aspects of health lifestyles” (Mollborn and Modile 2022). But previous scholarship has not systematically demonstrated how these aspects integrate to create health lifestyles or form in the interplay among parents, children, and community collectivities. We address each aspect in turn.

**Table 1.** Inductively Derived Components of Children’s Health Lifestyles.

Health Lifestyle Components
1. Understandings of health (parent) 2. Health behaviors (child) 3. Parenting narratives (parent) 4. Identities (parent and child) 5. Norms (community collectivities)

#### Understandings of health (parent)

The first component of children’s health lifestyles, understandings of health, undergirds the others. As described, our previous research (see Pace et al. 2022) has articulated these expansive understandings of children’s health that parents draw on to craft children’s health lifestyles, including behaviors, identities, narratives, and norms. Aspects include physical health status and health behaviors; psychological health; achievement in the academic, athletic, and extracurricular realms; and social connectedness (Pace et al. 2022). Sofia articulated such a multifaceted understanding: “I think everything has to be in balance to be healthy . . . I think it’s important to have friends, it’s important to exercise, it’s important to eat well, it’s important to . . . .” Her husband jumped in: “to know other cultures.” Similarly, Emma said, “Mental and emotional [health] is really important, so we kind of focus on stress, health, and healthy lifestyle.”

#### Health behaviors (child)

The previously empirically documented aspect of children’s health lifestyles is their health behaviors. Reflecting parents’ expansive understandings of health, we found that parents related a wide variety of child behaviors to health, including those typically related to physical health, like diet and exercise, but also those related to psychological well-being, academic achievement, and social connection. Brittany’s lifestyle described previously is one example, as are Linda and her 9- and 11-year-olds. Self-describing as “probably an average family, average parent,” Linda details her efforts to oversee a wide variety of health-related behaviors in her children, focusing on nutrition, sports participation, unstructured play, sleep, curbing behaviors that she fears can lead to food and technology addictions, and managing behaviors that she relates to psychological resilience, self-esteem, and the capacity to learn. We cannot rigorously investigate the frequencies of children’s health behaviors with our qualitative data, a task for future research.

#### Parenting narratives (parent)

As in Linda’s and Dan’s interviews, most parents link children’s health to thoughtful, well-articulated, health-oriented narratives around parenting. In our communities, intensive parenting—which falls mostly on mothers, although some fathers, like Dan, are heavily involved—entails constructing a narrative that justifies their child’s individually tailored health lifestyle. These narratives are similar to but more complex and multifaceted than the health lifestyle narratives class-privileged U.S. young adults (but not those less privileged) said they had learned from parents growing up (Mollborn and Modile 2022). Reflecting other trends toward the neoliberal individualization of lives (Reich 2016), parents do not simply adopt available cultural templates for children’s lifestyles. Rather, customized parenting choices and narrative justifications are expected, reflecting parents’ unique identities. Resource constraints on health lifestyle construction are mostly invisible in parents’ narratives.

Andrea says her parenting is informed by “a lot of reading” because “you just want them to be healthy.” Andrea’s narrative highlights the “constant struggle” of balancing children’s “mental,” “physical,” and “spiritual” health. She focuses on “the routines and their structure and how you talk to them and how you get through those hard days and nights where everything is just a nightmare. It’s so key. And it really does pay off.” She responds when asked if her 8- and 11-year-olds are healthy: “Yeah. They have friends, they go to birthday parties, they’re active. Thank God they don’t have ongoing issues with dyslexia or learning disorders. I’m blessed. I think they’re both a little overweight, but I’m sure that will even out.” She went on to discuss her efforts to “open up a conversation” with her daughter (but not her son) about balancing her desire to be sedentary and create art with the need for cardiovascular activity. Andrea’s narrative presents her as a health-focused mother who applies hard work and discipline to parent successfully. Parents’ narratives complement behaviors and subjective understandings of health as key aspects of children’s health lifestyles in these class-privileged communities.

#### Identities (parent and child)

A child’s health lifestyle reflects parents’ identities, represented through behaviors and narratives in a public way that is appraised by other community members. But potential judgment is not the only reason parents’ identities are highly invested in constructing lifestyles: Parents view health lifestyles as crucial for shaping children’s well-being and success. Parent and child identity expression through children’s health lifestyles is normatively prescribed in these communities: Parents should determine the best lifestyle to reflect their identities, and it should also be portrayed as aligned with the child’s identity.

Parent identities are palpable throughout their narratives, such as Andrea’s portrayal of her hard work to parent successfully around health and Dan’s self-promoting comparisons of his boundary-setting efforts to make Brittany’s lifestyle healthier. Similarly, Dawn discusses at length how her identity influences her parenting of her 9- and 5-year-olds, from fostering resiliency and “a deep sense of self-worth and self-love” to instilling respect and an understanding of the importance of eating nutritiously. Her narrative repeatedly cites outside evidence that her children’s identities are developing along these lines, from them happily drinking green smoothies to a neighbor commenting on how “comfortable,” “gracious,” and “well adjusted” they are.

Dawn’s attention to her children’s identity expressions is typical, but parent and child identity expressions often conflict. Children, by expressing their own preferences, disrupt the smoothness of parents’ attempted socialization into lifestyles. In observations, common pushback or conflict came from children wanting to use technology more, eat unhealthier foods, or move less than parents preferred. Parents and children struggling for control of children’s behaviors caused tensions. Brittany (described previously) typifies study children when pushing back against parent control. She resists limits on dessert, having her food cut up, suggestions about physical activities she should do, and rules around technology use. Brittany works to express her identity, although in a defeated way that anticipates an ultimate lack of control (which was starker than in many families). These realities of child identity expressions complicate the neater narratives parents often present, such as Dan’s accounts of Brittany’s compliance.

Further complicating these dynamics, many parents in our sample articulate a norm that a health lifestyle should reflect the child’s identity. Parents often work hard to make children’s behaviors both fit their parent identities and credibly appear to reflect the child’s identity. Christine describes the intensive parenting efforts involved in accommodating 10-year-old Noah’s preferences:

It took us a long time to find something Noah wanted to do. When he was younger we had him in gymnastics, tae kwon do, swimming. We tried everything until we hit soccer, and then he just loved it. So he really didn’t want to do anything else until he hit basketball. And so now his entire focus is basketball and soccer. And that’s what he wants. . . . So all that is his choice.

Christine describes Noah’s sports involvement as “his choice,” but Noah’s agency was actively constructed and constrained by his parents repeatedly enrolling him in sports until he found two he enjoys. Noah is being pressured to “prefer” a health lifestyle that involves “loving” playing multiple sports. He likely understood that his parents’ efforts to put him on sports teams would not cease until he chose two sports, which constrains his identity expression even as Christine portrays him as choosing.

It would have been far easier for Christine simply to choose two sports, so she is expending considerable effort to encourage Noah’s identity expression. Thus, even though children’s identity expression often causes problems for children’s enactment of their parents’ preferred behaviors, parents encourage it as an important facet of middle-class health lifestyles. Participants differ, however, in how highly they prioritize children’s identity expression (see the following).

#### Norms (community collectivities)

Community, or collectivity, norms are the final component of children’s health lifestyles that we identified. They regulate what is acceptable in other aspects and are tools used by parents to reinforce lifestyles. Many parents explicitly rely on community norms to reinforce their chosen lifestyle. Nick describes health-related norms in Greenville as a draw for moving there, such as valuing “exercise and organic and growing food in your backyard . . . I feel like my fifth grader, after being in a Greenville school, has been fairly indoctrinated about the food, which I never was growing up. You know, the food groups, and creating a healthy meal, and eating different colors when we’re eating, and the importance of that.” Nick feels that strong norms in his son’s school reinforce his health lifestyle.

Yet community norms can threaten chosen health lifestyles, such as when an “achievement of independence” lifestyle is condemned by other parents (see the following). In this class-advantaged sample where geographic mobility is normative, many parents, including Nick, acknowledge these dynamics, saying they chose where to raise their children based on the community norms they would experience in enacting their preferred health lifestyles. This exemplifies the formation of “overrider enclaves” resisting the “default American lifestyle” posited by Mirowsky and Ross (2015). Thus, parent preferences can shape the community norms in the child’s lifestyle. Hector says of Greenville’s norms that encourage healthy eating and physical activity, “We brought them [the children] to the environment where the values we valued are there. And they are getting them, not just from us, which in hindsight has been amazing. But it’s sort of what brought us here, right?” Although she can feel judged for her lifestyle approach (see the following), his wife, Sofia, echoes, “I feel we have a lot in sync with other parents and the way they teach their children here.” Many parents note synergies or tensions between community and family in fostering health lifestyles.

Parents sometimes acknowledge that community resources facilitate norms. A Greenville focus group parent described the community as “family oriented.” Another parent tentatively linked that to resources: “I wonder if it has something to do with that it’s a more affluent community, and so, you know, there’s more free time maybe, or more ability for parents to be involved because they’re not trying to drag all of their jobs.” The first parent concurred, and a third noted that “all that land”—public open space—contributed to community cohesion and norms. Parents’ understanding of community aspects of children’s lifestyles is often nuanced, articulating selection and causation and relating resources to norms.

### Localized Health Lifestyle Options

Understandings of health, behaviors, narratives, identities, and norms together comprise children’s health lifestyles, but the lifestyles’ actual content varies within our sample. As health lifestyle theory suggests, community norms prescribe that parents should intensively construct a deliberate lifestyle for their children from among acceptable options. Before articulating differences, we emphasize that these middle-class communities’ predominant lifestyles have important similarities in health-related norms: (1) a strong focus on nutrition, exercise, and academic achievement; (2) intensive parenting efforts required to sustain the lifestyle; and (3) the high salience of the child’s future for their health lifestyle.

We summarize the four prevalent health lifestyles in our communities, primarily illustrated using four families. Table 2 articulates the lifestyles and the two dimensions that differentiate them: the relative importance of parent versus child identity expression and the relative focus on the future versus present. Although most parents blend elements of multiple health lifestyles in constructing a lifestyle, most children’s lifestyles primarily fall into one type. We argue that the interweaving of different health lifestyle components (understandings of health, behaviors, narratives, identities, and norms) strengthens the power of these lifestyles, likely making them more effective for transmitting class advantage into adulthood than a lifestyle solely composed of behaviors would be.

**Table 2.** Typology of Childhood Health Lifestyles and Selected Associated Behaviors and Parent Narratives Documented in Our Middle- to Upper-Middle-Class Communities.

	Whose Identity Expression Is Prioritized?		Prevalent Behaviors and Attitudes
	*Parent*	*Child*	
Temporal focus? *Long-term*	Elite achievement lifestyle	Achievement of independence lifestyle	Behaviors: more structured exercise, less unstructured play, more (implicit) body regulation Narratives: competencies/achievement focus
*Short-term*	Family connection lifestyle	Let kids be kids lifestyle	Behaviors: more unstructured exercise and play, less body regulation Narratives: well-being/stress focus
Prevalent behaviors and attitudes	Behaviors: more restricted technology use Narratives: focus on parents developing child identity, parents feeling less alone and more successful	Behaviors: less restricted technology use Narratives: focus on child developing own identity, parents feeling more alone and worried about being successful	

We expect that different communities likely contain different prevalent children’s health lifestyles because societal notions around parenting intersect with community demographics and localized cultures (see Brown-Saracino 2015). Our communities’ cultural focus on physical health, athleticism, and outdoor activities shapes health lifestyles, potentially making them more salient for parenting than elsewhere and attracting new families who seek such lifestyles. Family and community resources are also important for enabling these lifestyles. The predominant lifestyles that we identify likely appear in some other communities but are presumably joined by additional lifestyles yet to be identified.

#### Elite achievement

The most prevalent, normatively dominant, and typically most resource-intensive health lifestyle for community children, the “elite achievement” lifestyle, combines a future-oriented focus on skill building with high levels of parental identity expression in everyday interactions (see Table 2). Achievement is part of parents’ understandings of child health (Pace et al. 2022). Parents from upper-middle-class childhood backgrounds were disproportionately represented in this health lifestyle. The elite achievement lifestyle has much in common with Lareau’s (2011) “concerted cultivation” parenting style, which involves heavily structured time spent on extracurricular activities and school achievement. Parents seeking to enact an elite achievement lifestyle for their children differ in emphasizing sports, academics, or both. Unstructured playtime and technology use are limited. They view their child’s current success, including in achieving a fit body, as linked to future well-being and sometimes compromise children’s current health and stress levels to achieve these goals (Mollborn et al. 2021). However, like parents in other lifestyles, they tend to view it as expressing their children’s preferences and making them happy. Elite achievement parents feel that they are crafting a “normal” health lifestyle for their child and tend to view their parenting as relatively successful.

For example, Laura and 10-year-old Jacob are from a White, upper-middle-class Greenville family. Laura says, “I feel like so much of our peer group is pretty homogeneous” in their parenting and that she follows the dominant norm. Jacob is “very active,” playing on multiple sports teams many seasons and doing supplemental academic work. Laura emphasizes Jacob’s identity expression in his lifestyle: “My ultimate goal for my son is really just to have fun with his activities.” She also links Jacob’s extracurricular involvement to future success: “If you don’t get in on the ground floor in some of these sports, you’re never going to have a shot at playing in college.” Laura worries about the academic implications of Jacob’s problematically “too easy” workload, although he enjoys having little homework. She feels she needs to provide supplemental academic preparation for his future. Finally, like parents in other health lifestyles, Laura connects Jacob’s health lifestyle to parent and child identities: “We’ve just always gone really hard. Like, I just go really hard. Their dad goes really hard. And that’s just kind of the way we raise them. Yeah, a rolling stone gathers no moss.” Similarly, Kaya enrolled a sometimes reluctant 9-year-old Madeline in multiple sports to combat overweight and her physical tendency to “do the minimal amount of work necessary” while also being “extremely involved” in fostering Madeline’s academic achievement and interest in nutritious cooking.

#### Achievement of independence

Like elite achievement, the “achievement of independence” health lifestyle focuses on the future and achievement, but it emphasizes child identity expression in everyday activities, working toward a distinct goal: the child’s independent acquisition of skills and maturity to facilitate successful adulthood. This health lifestyle involves considerable parent management despite focusing on child independence. Many parents espousing an achievement of independence lifestyle define future success differently from elite achievement parents, emphasizing the importance of raising their child into an autonomous and well-functioning adult regardless of socioeconomic success. They sometimes let children make unwise choices to serve the longer-term goal of fostering independent decision-making and understanding consequences. Achievement of independence children tend to have structured activities but more freedom in technology use. Parents typically feel more alone in their parenting choices.

Jasmine, Aaron, and 9-year-old Evie belong to a multiracial working- to middle-class Springfield family. Aaron said, “We are super intentional with what we do with the kids.” Evie does more chores than most study children, often toward a stated goal of fostering adult skills. When Evie asks Aaron whether she is “done now” with toothbrushing, Aaron responds, “You’re getting to the age where you can decide if you’re done or not. Like, you can’t keep on coming to me as an adult and be like, ‘Is two minutes long enough to brush?’ . . . I mean, you have to answer these questions for yourself.” Whether Evie brushes her teeth long enough today is secondary to the future-oriented goal of independently enacting appropriate health behaviors. Jasmine is clear about the lifestyle they foster: “I feel like we are ‘free range,’ if you want to put labels on all these things. We really give our kids the opportunity to learn by themselves. Not necessarily alone, but I’m going to watch you try that and see if you can do it.” Aaron articulates their goal of future independence and competence: “I want her to be confident. I want her to be able to choose college if she chooses. I want her to choose to travel if she wants to.” They feel on track with meeting these goals because “I feel like she’s healthy in her head, where she knows pretty much who she is.”

Yet Jasmine and Aaron sometimes feel the sting of others’ judgement, suggesting that achievement of independence is less normative than elite achievement. Describing an outdoor interaction with a hovering parent, Aaron says, “If I see you on top of your kid, it’s not a big deal to me. But if they see us letting the kids go, they definitely have to say something [negative to me]. I just don’t understand that.” Similarly, Sofia schedules many activities for her children and seeks to instill hard work, but when they are with other children, if “they’re not getting into really dangerous situations, I let them be.” She thinks friends judge her for “giving our children too much freedom” and says “you get that little smirk” from other parents.

#### Family connection

The third prevalent health lifestyle combines the focus on parent identity expression that is similar to elite achievement with a new parenting dimension: emphasizing the child’s present over the future. Sometimes in explicit or implicit resistance to elite achievement, “family connection” parents prioritize current well-being and lower stress over future considerations, achieved by fostering social relationships, particularly with family. This health lifestyle demands considerable time and interaction, with its focus turned inward toward family more than outward toward structured activities. Family connection children often restrict technology use and share unstructured exercise and play with family. Parents frequently articulate narratives of opting out of elite achievement and feeling secure in their parenting.

Sharon and 9-year-old Finn are in a White, middle- to upper-middle-class Springfield family. Sharon’s family spends lots of time together engaging in healthy behaviors: “The more we can be outdoors together, the better. . . . We like to ski together as a family and do little trips together.” Finn plays on a traveling sports team. Although this activity could signal elite achievement, Sharon situates his involvement within their family connection lifestyle, saying, “I love [traveling overnight to games for] soccer because it has brought us together as a family.” Sharon restricts Finn’s extracurricular reading—supplemental academic preparation that could foster future socioeconomic success—when it threatens their “family time.” Like other family connection parents, Sharon directly links her parenting identity to family members’ healthy lifestyles:

I think being healthy is having enough fresh air in your day and having good food to eat, having exercise, having family time, having friend time. I feel that’s what I do in my life. For myself and my family, I try to puzzle together—like balance it out, so that we’re all getting nourished on all those levels. . . . And sometimes it’s unbalanced. So it’s like, okay, we need to have some family time . . . I kind of orchestrate it, I think, in our family.

Sharon carefully “orchestrates” balance in her family to improve current well-being and health, talking about “family time” as an antidote to stressors and imbalances. Similarly, Mark and Rachel curtail technology use and encourage joint physical activities to create more family time with their 7- and 9-year-olds. Rachel says, “The message is that you’re a part of this family, and you need to contribute. And that’s part of how we are great together. There’s this message that we work together, and it’s great, and we all need to help each other.” Although many family connection parents discuss opting out of some high-pressure goals for their children’s future to reduce stress, be healthier, and increase family interaction time, they view their health lifestyle as equally intensively constructed and challenging to maintain.

#### Let kids be kids

The fourth health lifestyle similarly focuses on present mental health and stress levels, but the child’s identity expression, more than the parents’, is considered crucial for well-being. Despite some similarities to Lareau’s (2011) working-class “accomplishment of natural growth” parenting style, “let kids be kids” parents intensively manage children’s lives, constructing the lifestyle to accommodate child preferences. Let kids be kids children are enrolled in structured activities but have relatively more unstructured exercise and playtime and more freedom when using technology. Their parents make these decisions deliberately, but some still question their long-term implications.

Anna and 10-year-old Chloe are in a White, upper-middle-class Greenville family. Anna articulates her parenting goal as inculcating “that feeling of, you’re okay just the way you are. . . . And everybody has a unique gift to offer, and you just have to figure out what it is.” These statements foreshadow the future but foreground current well-being and child identity expression. Anna says that like her, Chloe is “introverted” and “usually wants to just come home after school” instead of going to sports and playdates. Anna “puts [her children’s] preferences first,” but because Chloe has trouble making friends, Anna has decided to schedule one playdate a week with a friend of Chloe’s choosing. Chloe wants to join a traveling sports team that Anna thinks is “very intense, and I’m a little concerned . . . but she loves this team. So we’re just going to go for it.” Reflecting this lifestyle, Anna helps Chloe express her identity even when privately critiquing her choices. Yet Anna sounds conflicted when comparing herself to elite achievement parents—an indication that this lifestyle may be less normative in Greenville—worrying that her friends’ daughter “is going to, like, Princeton or something, and maybe I should be pushing more.”

Like Anna, Julie goes to great lengths to facilitate 9-year-old Callie’s identity expression. Callie participates in multiple sports, and their family focuses on nutrition, but self-expression is core to Julie’s narrative. She says, “It’s very, very important for Callie and I that she expresses herself. . . . We believe in self-control and self-regulating as a way of teaching her self-care. . . . It’s very important that she knows when she’s in a situation that is not okay, that she can speak up.” Reflecting this emphasis, Julie says Callie is allowed several “personal days” when she can choose to miss school, and there are no parental controls on family technological devices.

## Discussion

### Implications for Inequalities

This qualitative study innovates by documenting the components and prevalent types of child health lifestyles in two middle-class communities. Well-developed theory has posited that health lifestyles extend beyond behaviors and that people choose lifestyles from among options available by social status, but empirical scholarship has not documented whether or how this happens. Our study starts to bridge this gap, empirically unpacking the last box in Cockerham’s (2023) theoretical model, which contains the construct of health lifestyles and links it to social reproduction. We found that parents construct expansive understandings of “health” for their children (Pace et al. 2022), which undergird children’s health lifestyles that are further comprised of health-related behaviors, narratives, identities, and norms. Parents craft health lifestyles from among locally available options and in concert with child identity expression. Parents navigate potential judgments of their health lifestyle choices and articulate awareness of community norms that inform decisions. These lifestyles are likely important for reproducing families’ and communities’ social, socioeconomic, and health advantages.

Although our cross-sectional data cannot document long-term effects, parents imagine that their children’s health lifestyles matter for identities and future lifestyles, health, and socioeconomic attainment. We agree that this is likely. By prioritizing child versus parent identity expression and present well-being versus future competencies, parents feel that their lifestyle choices inform their child’s futures. Anna’s narrative of “trying to develop a person who is intrinsically motivated to do this stuff, not for me” drives her let kids be kids lifestyle approach. She is attempting both to shape Chloe’s behaviors and instill an identity that will presumably shape her future lifestyle. Similarly, Laura teaching her son to “go really hard” is about both elite achievement and a narrative around discipline and hard work, which can be used to justify advantaged class position (Luna 2019; Mollborn and Modile 2022). Such mastery of behaviors and self-presentations that match cultural logics of classed institutions (Gage-Bouchard 2017) is linked to socioeconomic attainment (Calarco 2018; Lareau 2011). Despite some differences, all health lifestyle types in our study communities are class-privileged and feature intensive parenting, socialization into entitlement, and development of thin bodies through diet and physical activity, which facilitate class distinctions and privilege in adulthood (Calarco 2014; Mollborn et al. 2021). Thus, constructing health lifestyles through behaviors, narratives, identities, norms, and understandings of health is a mechanism through which class and health distinctions can perpetuate intergenerationally and across life. Qualitative research comparing class-advantaged and -disadvantaged young adults has indeed found this mechanism to be salient (Mollborn and Modile 2022). Collecting more data directly from children and following them longitudinally could help articulate these processes.

### Implications for Health Lifestyles Research

This study’s effort to harmonize health lifestyles theory with extant empirical research has research implications. Most empirical health lifestyles work is quantitative, using nationally representative data and latent class analyses with specific health behaviors as inputs. This method identifies predominant configurations of inputs, which researchers label as prevalent lifestyles in a national population but cannot adjudicate whether each behavior should be part of the lifestyle or if behaviors or other components have been missed. This research has spurred many advances, such as identifying frequent combinations of healthy and unhealthy behaviors within individuals and articulating life course and network dynamics of health lifestyles (for a review, see Mollborn et al. 2021). More progress is needed in several areas.

First, parents’ expansive understandings of children’s health (Pace et al. 2022) justify incorporating a wider variety of health behaviors. For example, Cockerham’s (2023) expanded theoretical model articulates technology use and COVID-related social distancing as newly important behaviors for health lifestyles. Behaviors related to psychological well-being, social connection, and academic achievement could also be included, according to our parent participants. Latent class analysis frameworks can accommodate broad operationalizations.

Second, both theory and our analysis suggest that health lifestyles research should operationalize lifestyles more broadly beyond behaviors. We found that health lifestyles combine understandings of health and health-related behaviors, identities, narratives, and norms (Cockerham 2023; Krueger et al. 2009; Mollborn and Modile 2022). Without this expansive conceptualization, efforts to understand health behaviors or parenting practices may lack important context and be difficult to change through policies. Qualitative research combining public and private narratives with observations of behaviors and interactions, as we have done, is promising for articulating these processes. But quantitative research can also make strides. On a related topic, Lankes (2022) conducted latent class analyses identifying prevalent U.S. intensive parenting approaches that incorporated a variety of behavioral and attitudinal measures from survey data. Behaviors and attitudes combined in interesting ways that furthered theoretical understandings. We argue that appropriate operationalizations of health lifestyles depend on research context but should ideally be grounded in target individuals’, rather than researchers’, conceptualizations of health. These understandings can set boundaries around a study’s operationalization of health lifestyle by guiding the selection of behaviors for inclusion and possible measures of norms or attitudes, identities, and narratives depending on the method.

Third, in moving beyond behaviors, research can broaden beyond individuals to examine families and communities or collectivities. Situating individuals’ agency within linked lives is important yet overlooked (Landes and Settersten 2019). And health lifestyles are by definition collective (Cockerham 2005; Frohlich and Potvin 1999), an empirically neglected aspect. We found that parents, children, and communities were all fundamental to components of children’s health lifestyles. Although a few studies have examined collective influences on individuals’ health lifestyles, future research still needs to move beyond the individual level to show how lifestyles themselves and their structure–agency interplay are collective.

Fourth, this study joins a few qualitative and quantitative investigations (e.g., adams et al. 2021; Lee et al. 2015; McGarrol 2020) in identifying localized health lifestyle options. We innovate in exploring how people negotiate choosing among options, but more research is needed. Almost nothing is known about how local health lifestyle options vary systematically or intersect with individuals’ characteristics when forming a person’s health lifestyle. Most extant research is conducted nationally, but theories expect health lifestyles to play out locally. This means that many aspects of our findings likely do not apply in socially disadvantaged settings. For example, Eriksen et al. (2024) and Mollborn and Modile (2022) found that narratives about health lifestyles linked to strongly health-focused identities were prevalent among young people from class-advantaged but not class-disadvantaged backgrounds. Health lifestyle options should be examined using quantitative data that include respondents clustered within widely varying communities, and qualitative research should document a diversity of local communities. Eriksen et al’s (2024) longitudinal qualitative study in Norway is one such step.

We argue that moving health lifestyles research in these directions, using a variety of methods, can more fully articulate the power of health lifestyles for understanding how advantage is perpetuated across lives and generations and how health lifestyles themselves are a tool that creates and props up inequalities (Korp 2008). Class-advantaged children’s health lifestyles in our communities involve learning specific behaviors that community norms deem “healthy” and worthy of reward, which become embodied through thinness and fitness. They also entail developing identities organized around the fundamental importance of expansive understandings of health and health behaviors, which are expressed in narratives that link health to discipline and moral worth. It is this combination of characteristics, beyond specific health behaviors, that propels children toward advantaged futures.

Understanding health lifestyles in this way could spur more effective and appropriate policy efforts. Changing people’s health behaviors is notoriously difficult—in part because a target behavior likely combines with other behaviors within a health lifestyle that is also comprised of identities, narratives, norms, and understandings of health. Returning to Brittany, her behaviors have been carefully planned and justified by Dan and Mary, who relate their chosen lifestyle for Brittany to their own traditional, family-oriented identities and their moral worth as parents. Policy efforts from Brittany’s community or school to change one of her health behaviors are unlikely to succeed unless they engage with her broader health lifestyle, its links to both her present and future, her parents’ deep identity investment, her own identity expression, and the lifestyle’s embeddedness within localized community norms. A successful effort to change Brittany’s behavior must therefore move well beyond the individual child and her family—which is harder to implement but potentially much more effective. This empirical reality could translate into starkly different policy approaches to intervening in social inequalities and children’s health.
